# Glomangioma Supply from Profunda Femoris Artery in Peripheral Artery Disease

**DOI:** 10.3390/clinpract12050078

**Published:** 2022-09-18

**Authors:** Claudiu N. Lungu, Mihai Creteanu, Gabriel Olteanu, Aurelia Romila

**Affiliations:** 1 Department of Surgery, Clinical Country Emergency Hospital, 810249 Galati, Romania; 2 Department of Radiology, Country Emergency Hospital, 720001 Suceava, Romania; 3 Department of Surgery, Clinical Country Emergency Hospital, 400000 Cluj, Romania; 4 Department of Gerontology and Geriatrics, Clinical Country Emergency Hospital, 810249 Galati, Romania

**Keywords:** glomangioma, peripheral artery disease, profonda femoris artery

## Abstract

This is a case report of a 5.6 cm glomangioma supplied by the femoral profunda artery in a 66-year-old male patient with severe peripheral artery disease. The patient complained of discomfort and mild pain at the place of the lesion and an accelerated growth rate in the last two months. A nodular mass located laterally on the left foot, elastic, covered with a thin skin, and mobile, was noted on the clinical exam. Doppler exam demonstrated an active vascular supply. CT angiography showed a femoral profunda artery blood supply and a severe asymptomatic bilateral peripheral artery disease (PAD). The lesion was removed entirely by surgery. A microscopy exam revealed a glomangioma. After surgery, the patient recovered unevenly. However, the patient experienced wound-healing issues that resolved after four weeks of surgery. Although the patient’s PAD was severe, the lesion presented with a burst in dimensions weeks before surgery.

## 1. Introduction

Glomangiomas are benign skin tumors characterized by abnormal, smooth muscle-like glomus cells [1]. The tumors originate from transformed smooth muscle cells in specialized arteriovenous shunts in acral sites, especially the fingertips [2,3,4]. Glomangiomas appear in early life as small nodules situated deep in the dermis, scattered on the skin. They are not likely to be painful [5,6,7,8]. Glomangiomas have a tropism for the upper extremities and occasionally are found on the lower extremities, head, and back. Glomangiomas have a male predominance, while females are more frequently (in 70% of cases) found to have solitary glomus tumors [9,10]. Histopathologically, glomus tumors contain dilated vascular channels surrounded by glomus cells. The glomus cells are monomorphic round or polygonal cells with plump nuclei and scant eosinophilic cytoplasm [11,12,13]. Treatment is individualized to the patient and guided by the clinical presentation. Treatment is not always indicated, particularly in asymptomatic cases of glomangioma. Surgical intervention, when needed, is typically excision with primary closure [14,15].

## 2. Materials and Methods

The methods applied were anamnesis followed by physical examination, diagnostic imaging, morphophonological diagnostics, and surgery followed by a three month follow-up, respectively. Peripheral artery disease (PAD) was classified according to Fontaine classification, and oscillography was also performed.

## 3. Results

Anamnesis: The patient is a Caucasian, retired, 68-year-old male. Leg pain, paresthesia in the groin, and local erythema were the major complaints of the patient. A positive history of hypertension and gastric ulcer was noticed. The family medical history was unremarkable. Alcohol and tobacco consumption were also noted.

**Figure 1:**
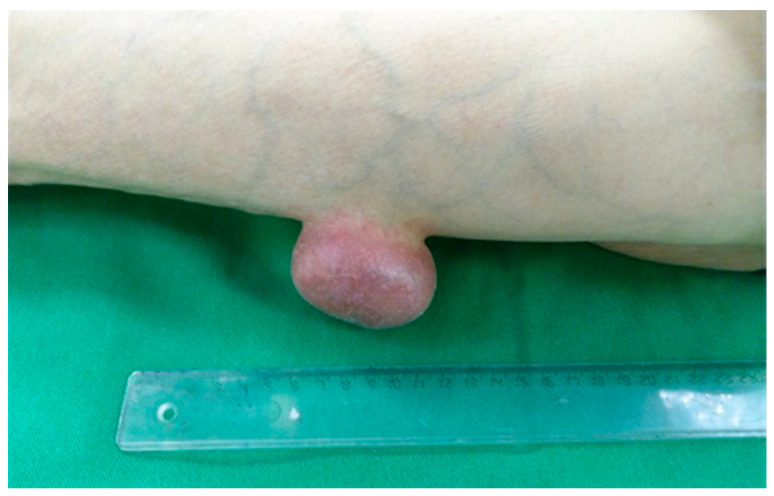
Left leg with a 5.6 nodular formation.

A physical exam showed a mild femoral pulse at the left groin, with a 5.6 cm pseudotumoral formation below the left knee (Figure 1). The structure was not adherent to the subcutaneous plane and had an elastic consistency. The patient recalled the presence of the tumor six years ago with continuous growth.

Diagnostics: The patient was found to have Fontaine type II A PAD with a measured 0.9 ABI for the right foot and 0.8 ABI for the left foot.

**Figure 2:**
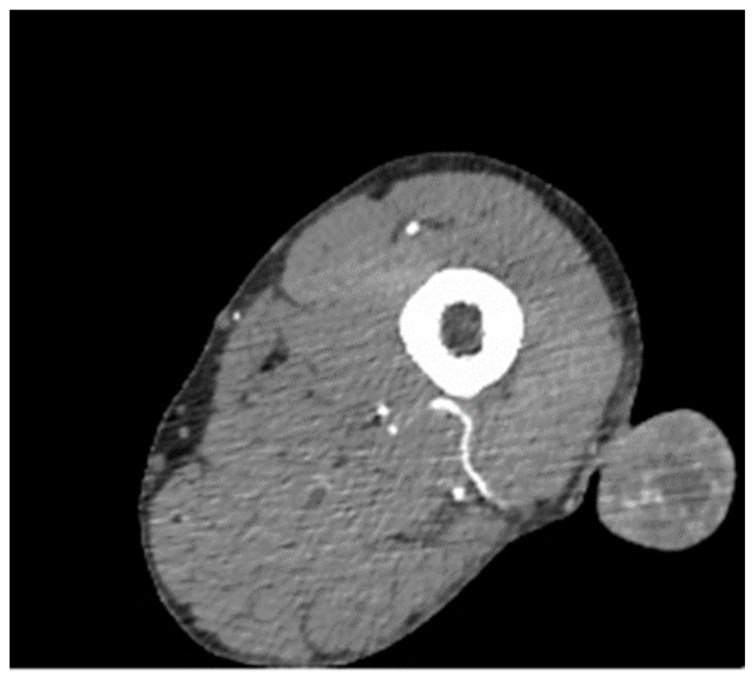
The CT angiographic scan of the lower limb shows the nodular formation with a vascular pedicle connected to the profound femoral artery. Blood vessels are also noticed inside (Supplemental Figure S1).

CT contrast imaging (pelvis and lower limbs) was performed to investigate the tumoral process further. (Figure 2).

**Figure 3:**
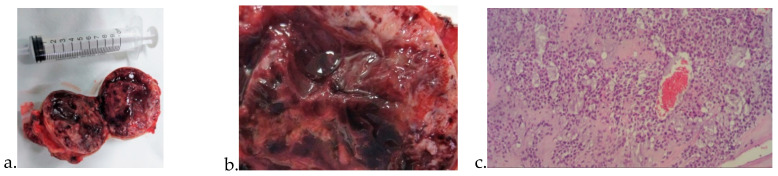
(**a**). Resected tumor showing various blood vessels, areas of necrosis, and clots. (**b**). Details of the resected tumor. (**c**). Histopathological exam offering dilatated venous channels with multiple rows of cuboidal glomus cells. Abnormal collections of glomus cells are noted. Unlike venous malformations, they present single or multiple rows surrounding cuboidal glomus cells. The glomus cells tested positively for vimentin and α-smooth-muscle actin. The three components needed for diagnosis were observed: glomus cells, blood vessels, and smooth muscle cells respectively: glomus cells: are small and uniform, round to oval shaped, with distinctive cell borders, centrally located punched-out nuclei, light eosinophilic or amphophilic cytoplasm; blood vessels: thin-walled, capillary sized, dilated; smooth muscle cells: elongated, mature looking.

The patient was subjected to surgery. The tumor incision was performed within tumor-free tissue. Jean L Bolognia et al.’s two-volume set described glomangioma clinical findings and therapy [16]. Another relevant work is the paper by Andrew L. Folpe that described 52 cases of atypical and malignant glomus tumors [17]. Glomangiomas with pedicles derived directly from main vessels (SFA) demonstrate extensive growth (over 5 cm). After the surgical excision, the morphophonological exam revealed the diagnosis of glomangioma (Figure 3).

Disease course: The morphopatolgical exam was remarkable for hemangioma. The patient tolerated the procedure well. A healing defect was observed three weeks after the incision. Dehiscence of the surgical incision was observed. The edges of the wound were no longer connected after suture removal. The defect was observed only in the skin and connective tissue and was not observed in muscles and profound planes. The defect was lined on 2 cm (a total of 5 cm). The healing deficiency was resolved surgically. After three weeks, the wound healed completely. Laboratory tests were standard except for a mild leucocytosis. No further diagnostic challenges were recorded.

## 4. Discussions

Although glomangiomas are relatively small tumors (1 cm), when the tumor vascular pedicle is connected to a large vessel, the cancer can probably reach a considerable size [18,19,20].

The differential diagnosis for glomangioma includes vascular malformation, which does not have perivascular glomus cell accumulation [21,22]. Other morphophonological entities included in the differential l diagnosis are myopericytoma, angioleiomyoma, and myofibroma. All of those have perivascular muscle cells but no glomus cells. In addition, Desmin positivity is noticed in this entity [23,24].

Malignant glomus tumors are rare. The criteria for diagnosing malignancy in glomus tumors are (a) tumor size of more than 2 cm and subfascial or visceral location; (b) atypical mitotic figures; (c) marked nuclear atypia and any level of mitotic activity; (d) and detection of Zimmerman’s pericytes [25,26].

Malignant glomus tumors are divided into three categories based on their histology: glomus tumors with local infiltration (LIGT), glomangiosarcomas arising in benign glomus tumors (GABG), and de novo arising glomangiosarcomas (GADN) [27].

Some instances of malignant glomus tumors are described. However, metastases are rare. The misdiagnosis of many of these lesions, such as hemangiomas or venous malformations, makes a precise diagnosis difficult. Vascular malformations do not have the perivascular glomus cell accumulations seen in glomangioma, Myopericytoma, angioleiomyoma, and myofibroma. All have variable degrees of perivascular muscle cells, but these cells do not have the classic cuboidal glomus cells aspect [28,29]. These tumors usually show a degree of desmin positivity in the perivascular cells, which is not seen in glomangioma. The incidence of glomus tumors is unknown. Multiple variants are rare, accounting for less than 10% of all cases. Glomangiosarcomas are extremely rare and usually represent a locally infiltrative tumor. Metastases do occur and are generally fatal. Surgery is the preferred treatment for benign glomus tumors [30]. The majority of glomus tumors are benign. They are usually located superficially (on or just below the skin, as in this case) and produce pain, swelling, and skin discoloration. Some of them may also be located deeper inside the body tissues. The exact cause of developing glomus benign glomus tumor is not determined, even if several genetic mutations have been detected. Most commonly, young adults are affected by these benign tumors, in contrast with the age of the presented patient. The diagnosis of benign glomus tumor is performed by clinical examination and biopsy. No major complications are reported. Surgical tumor removal is the most effective treatment for tumors with significant signs and symptoms. The prognosis of benign glomus tumors with appropriate treatment is excellent.

Glomus tumors can present a significant size if the tumor vascular pedicle is connected to a major artery, even if the patient presents with peripheral occlusive disease. In this case, the patient presented with intermittent claudication at 400 m. PAD was located on the common femoral artery bilaterally and the profunda femoris artery on the left leg.

## 5. Conclusions

A 5.6 cm glomangioma was diagnosed on a 66-year-old PAD patient. Glomangiomas of the lower limb can coexist with peripheral artery disease. Presumably, the morphology and tumor evolution do not depend on the arterial disorder’s extent and staging.

## Data Availability

Imaging dats is available on demand.

## Supplementary Materials

The following supporting information can be downloaded at: https://www.mdpi.com/article/10.3390/clinpract12050078/s1, Figure S1: CT scan of lower limb demonstrated glomangioma blood supply.
