# Supplementary figures and images for: Glomangioma Supply from Profunda Femoris Artery in Peripheral Artery Disease
Source: Clin Pract. 2022 Sep 18;12(5):755–9. doi: 10.3390/clinpract12050078 (PMC9498625; doi:10.3390/clinpract12050078)

Supplemental material S1: CT scan of lower limb demonstrated glomangioma blood supply

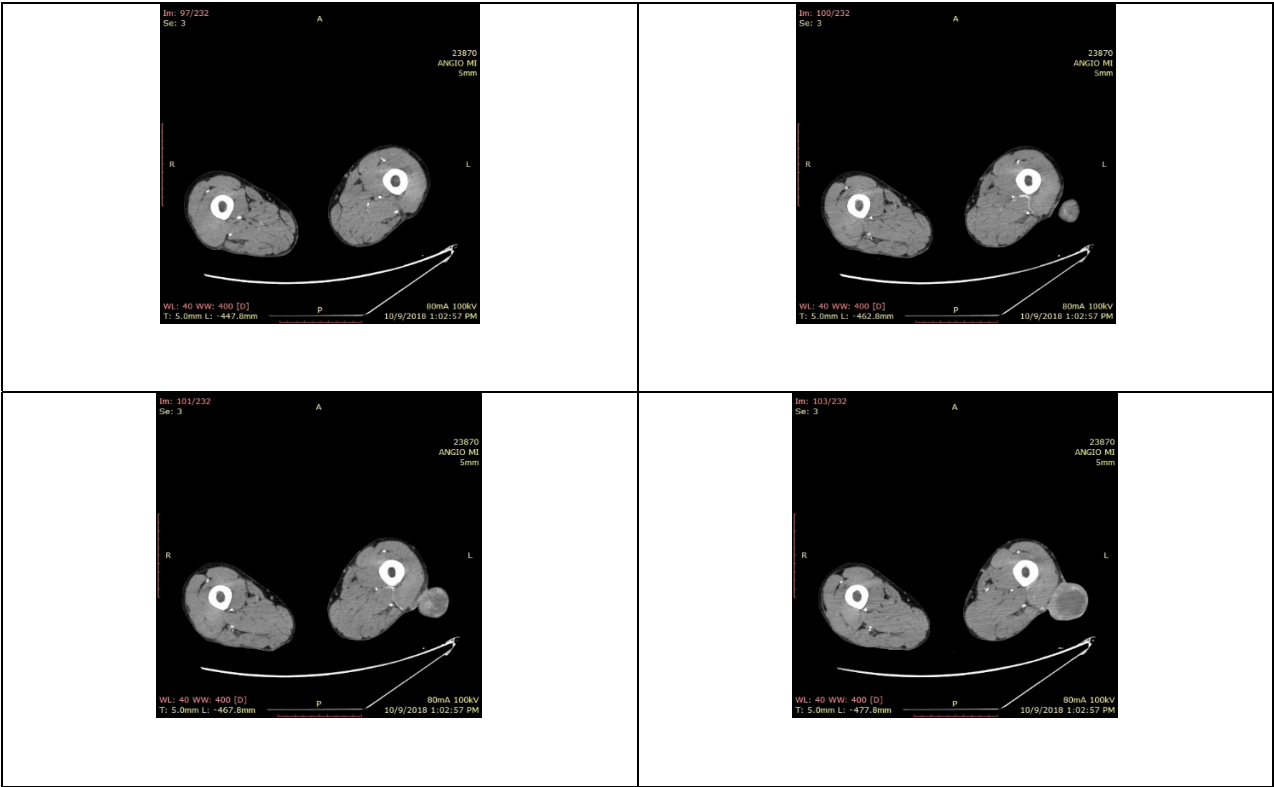

Supplement: Supplementary file 1 [file clinpract-12-00078-s001.zip › clinpract-1833375-supplementary.pdf]
